# Acute Pancreatitis in Children: Analysis of 15 Cases

**DOI:** 10.7759/cureus.90167

**Published:** 2025-08-15

**Authors:** Maria Rkain, Abir Azirar, El Bassil Karim, Amal Hamami, Aziza Elouali, Abdeladim Babakhouya

**Affiliations:** 1 Department of Pediatrics, Faculty of Medicine and Pharmacy of Oujda, Mohammed I University of Oujda, Oujda, MAR; 2 Pediatric Gastroenterology, Centre Hospitalier Universitaire Mohammed VI de Oujda, Oujda, MAR; 3 Pediatric Service, Centre Hospitalier Universitaire Mohammed VI de Oujda, Oujda, MAR; 4 Pediatrics, Centre Hospitalier Universitaire Mohammed VI de Oujda, Oujda, MAR; 5 Department of Pediatrics, Mohammed VI University Hospital, Faculty of Medicine and Pharmacy of Oujda, Mohammed I University of Oujda, Oujda, MAR

**Keywords:** abdominal pain, acute pancreatitis, lipase, pancreatic necrosis, pancreatic pseudocyst

## Abstract

Acute pancreatitis (AP) in children, although rare, has shown an increasing incidence. This retrospective study analyzed 15 pediatric AP cases over a six-year period from January 2019 to December 2024 at Mohammed VI University Hospital in Oujda, Morocco. The results revealed a male predominance with a sex ratio of 1.5 and a mean age of 8.8 years. The average time from symptom onset to consultation was 3.5 days. The predominant symptom was constant abdominal pain (100%), followed by vomiting (71.4%). Biologically, lipase levels were elevated in 100% of cases, with amylase elevation observed in 14.3%. Radiologically, patients were classified using the Balthazar staging system, with 40% at stage E. Etiologies were mainly idiopathic (26.6%), traumatic (20%), and biliary (20%). All patients received medical treatment, with 6.6% requiring interventional drainage. The majority had favorable outcomes, with 6.6% developing pseudocysts. Diagnosis relies on clinical, biochemical, and radiological criteria, and management is primarily supportive. Early and adequate treatment is essential to prevent complications. Antibiotic use and fluid management protocols require clearer definition.

## Introduction

Acute pancreatitis (AP) is an inflammatory condition caused by autodigestion of the pancreas and occasionally adjacent organs due to premature activation of pancreatic enzymes. This can provoke local and systemic inflammatory responses, including systemic inflammatory response syndrome (SIRS), potentially resulting in multiorgan failure and death [1]. Although AP is rare in children, its incidence has increased over the last two decades, nearing that seen in adults [2,3]. Unlike adults, where gallstones and alcohol abuse are the main causes, pediatric AP is often idiopathic, with trauma and congenital malformations also reported [1,4,5]. Diagnosis can be challenging due to variable presentations and low prevalence in children [1]. Serum lipase remains the most sensitive biological marker for diagnosis [1,4]. Imaging, especially ultrasound and contrast-enhanced CT, plays a pivotal role in confirming diagnosis and assessing severity [1,5]. Prognosis depends on the development of local or systemic complications and organ dysfunction [1,4]. Management is mainly supportive, although surgery may be indicated for drainage of fluid collections or treatment of biliary pathology [5].

The primary objective of this study is to evaluate the clinical, biological, and imaging features of AP in children at a tertiary care center. Secondary objectives include identifying common etiologies, describing management strategies, and documenting complication rates and patient outcomes.

## Materials and methods

This retrospective observational study was conducted from January 2019 to December 2024 in the Pediatric and Pediatric Surgery Departments of Mohammed VI University Hospital in Oujda, Morocco.

We included children under 16 years diagnosed with acute pancreatitis based on at least two of the following: characteristic abdominal pain, serum lipase >3 times normal, and imaging consistent with pancreatitis. Patients over 16 or without confirmatory criteria were excluded.

Data were collected retrospectively from medical records using a standardized MS Excel database (Microsoft Corp., USA), including demographics, clinical features, lab tests, imaging findings, etiology, treatment, and outcomes.

Ultrasound was the first-line imaging; CT scans were done for severe cases or suspected complications. CT was performed with standard abdominal protocols and reviewed by two pediatric radiologists using the Balthazar severity index.

Statistical analysis was descriptive using IBM SPSS Statistics for Windows, version 23.0 (released 2014, IBM Corp., Armonk, NY). Continuous variables are shown as mean ± SD; categorical variables as counts and percentages. No inferential tests were done due to the small sample size.

The study was approved by the hospital's ethics committee, with a waiver of informed consent due to its retrospective design.

## Results

In our series, 15 patients were included, averaging three cases per year. The mean age was 8.8 ± 3.8 years (range 3-15). There was a male predominance with nine boys (n = 9, 60%) and six girls (n = 6, 40%), with a sex ratio of 1.5. Most patients were from urban areas (n = 13, 86.6%).

The average delay from symptom onset to consultation was 3.5 ± 2.5 days (range 1-11). The most frequent presenting complaint was emetic epigastric pain (n = 7, 46.6%), followed by isolated epigastric pain (n = 4, 26.6%). Other presentations included febrile epigastralgia (n = 1, 6.6%), diffuse abdominal pain with vomiting and diarrhea (n = 1, 6.6%), and pain localized to the left iliac fossa (n = 1, 6.6%).

Regarding personal and family history, three patients had trauma (n = 3, 20%), and one patient (n = 1, 6.7%) was being followed for specific conditions such as acute lymphoblastic leukemia, macrophage activation syndrome related to Chediak-Higashi disease, or cystic dilation of the biliary tract. A family history of acute pancreatitis was reported in one patient. In addition, two patients (n = 2, 13.3%) had taken specific medications: one an appetite stimulant and the other a patient being treated for acute lymphoblastic leukemia (ALL), receiving chemotherapy sessions with L-asparaginase. Finally, one patient received the second dose of the Pfizer COVID-19 vaccine 18 days before the onset of symptoms.

On physical examination, abdominal tenderness was present in all patients, primarily located in the epigastric region (13 patients, n = 13, 86.6%), peri-umbilical area (three patients, n = 3, 20%), and right iliac fossa (one patient, n = 1, 6.6%).

Biologically, serum lipase and amylase were frequently elevated, with lipase levels exceeding seven times the normal range in six cases (n = 6, 40%). The average value was 10 times the normal level, with extremes ranging from 1.5 to 29 times the normal (Table 1). Other biological abnormalities included elevated CRP in all patients, leukocytosis with a predominance of neutrophils in 11 patients (n = 11, 73.3%), and hyponatremia in seven patients (n = 7, 46.6%).

**Table 1:** Lipasemia and interpretation for each patient. Lipase levels are given in U/L and are compared to the normal range (N). The interpretation indicates how many times the lipase value exceeds the normal range (×N). N: normal upper limit of serum lipase (75 U/L). Data are expressed as absolute values (U/L) and interpreted relative to the normal threshold. No statistical comparison was made due to the descriptive nature of this data.

Patient	Lipasemia (U/L)	Interpretation
Patient 1	112	1.5 × N
Patient 2	207	2.7 × N
Patient 3	247	3 × N
Patient 4	275	3.5 × N
Patient 5	322	4 × N
Patient 6	345	4.6 × N
Patient 7	420	5.5 × N
Patient 8	450	6 × N
Patient 9	486	6.5 × N
Patient 10	802	10.5 × N
Patient 11	900	12 × N
Patient 12	936	12.5 × N
Patient 13	1485	20 × N
Patient 14	1550	20.5 × N
Patient 15	2178	29 × N

**Figure 1:**
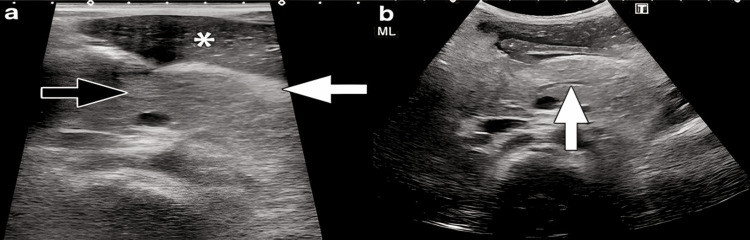
Transverse ultrasound images of the pancreas in a three-year-old boy with acute pancreatitis. Ultrasound was performed two days after the onset of an acute pancreatitis episode. a) The ultrasound shows a mildly bulky pancreas (black arrow) and echogenic, thickened peripancreatic fat reflecting edema (white arrow). b) Dilatation of the main pancreatic duct in the body of the pancreas.

In terms of radiological assessment, seven patients underwent abdominal-pelvic ultrasound. In three cases, the pancreas was enlarged, and in two others, it showed a heterogeneous structure (Figure 1).

**Figure 2:**
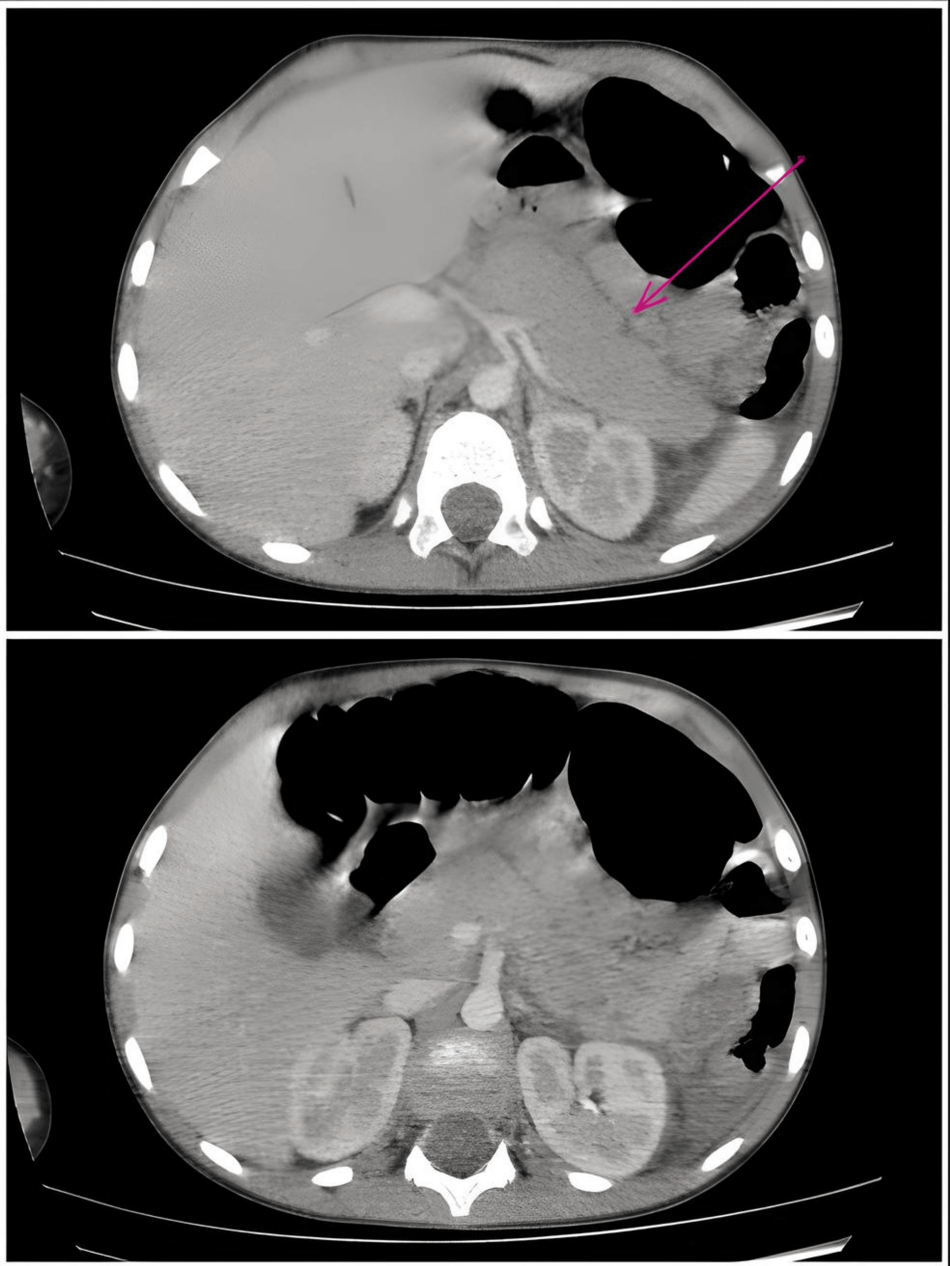
Axial slices of an abdominal CT scan performed in a five-year-old boy with acute pancreatitis, Balthazar grade E.

Abdominal CT revealed that ascites was the most frequent complication (n = 10, 66.6%), followed by pancreatic enlargement (n = 8, 53.3%) and the presence of pancreatic necrosis (n = 7, 46.6%). Peripancreatic fat infiltration was noted in six patients (n = 6, 40%). More rare findings included biliary dilation, pancreatic fracture, and spleno-mesenteric thrombosis, each observed in one patient (n = 1, 6.6%). According to the Balthazar CT staging, patients were classified as stage E (n = 6, 40%), stage D (n = 1, 6.6%), stage C (n = 2, 13.3%), stage B (n = 2, 13.3%), and stage A (n = 2, 13.3%) (Figure 2).

The etiological workup was performed in four patients (n = 4, 26.6%), revealing abnormalities such as sludge in the gallbladder, asymmetry of intrahepatic biliary ducts (IHBD), cystic dilation of the main and intrahepatic biliary ducts, and thickening of the terminal ileum suggesting inflammation, with a posterior horseshoe fistulous tract favoring Crohn's disease. An upper gastrointestinal endoscopy was performed in only one patient (n = 1) as part of this workup. The etiologies identified in our series were as follows: idiopathic in four cases (n = 4, 26.6%), traumatic in three cases (n = 3, 20%), biliary in three cases (n = 3, 20%), lithiasic in two cases (n = 2, 13.3%), malformative (cystic dilation of the biliary ducts) in one case (n = 1, 6.6%), systemic in one case (n = 1, 6.6%), drug-related in one case (n = 1, 6.6%), hereditary in one case (n = 1, 6.6%), post-COVID-19 vaccination in one case (n = 1, 6.6%), and post-measles in one case (n = 1, 6.6%) (Table 2).

**Table 2:** Etiologies of acute pancreatitis

Etiology	N	%
Idiopathic	4	26.6%
Traumatic	3	20%
Biliary	3	20%
Lithiasic	2	13.3%
Malformative	1	6.6%
Systemic	1	6.6%
Drug-related	1	6.6%
Hereditary	1	6.6%
Post-COVID-19 vaccination	1	6.6%
Post-measles	1	6.6%

All patients (n = 15) received symptomatic treatment, with a systematic average length of hospitalization of nine days (range 3-24 days). Digestive rest was prescribed for all patients (n = 15), with an average duration of 3.5 days (range 2-5 days), and all also received basic rationing. Regarding specific treatments, all patients (n = 15) were administered a level I analgesic (paracetamol and/or nefopam). Antispasmodic treatment with phloroglucinol-trimethylphloroglucinol was prescribed in seven cases (n = 7, 46.6%), and proton pump inhibitors (PPIs) were administered in six cases (n = 6, 46.6%). Antiemetics (metoclopramide or metopimazine) were given to three patients (n = 3, 20%). Furthermore, seven patients (n = 7, 46.6%) received antibiotic therapy, including six with stage E acute pancreatitis and one with a renal abscess; antibiotics included amoxicillin-clavulanic acid, ceftriaxone, metronidazole, imipenem/cilastatin, and amikacin. Ultrasound-guided drainage of pancreatic pseudocysts was performed in one patient (n = 1, 6.6%), but no surgical interventions were required (Table 3).

**Table 3:** Treatments administered

Treatment	N	%
Level I analgesics (Paracetamol and/or Nefopam)	15	100%
Antispasmodics (Phloroglucinol combination)	7	46.6%
Proton pump inhibitors (PPIs)	7	46.6%
Antiemetics (Metoclopramide/Metopimazine)	3	20%
Antibiotic therapy	7	46.6%
Ultrasound-guided drainage	1	6.6%

Clinical monitoring included regular checks of vital signs and general symptoms, as well as repeated abdominal examinations. The average time to pain resolution was 2.2 days (approximately 52.93 hours), with a range from one to five days.

Biologically, a decrease in lipase levels was observed in 12 patients (n = 12, 80%), while two patients (n = 2, 13.3%) had increased levels. Lipase normalization before discharge occurred in six cases (n = 6, 40%), while in two patients (n = 2, 13.3%), lipase levels at discharge were higher than at admission; one had traumatic acute pancreatitis and the other was associated with Crohn's disease. Radiological monitoring relied mainly on ultrasound, with CT used in some cases for diagnostic confirmation. The overall prognosis was favorable in 14 cases (n = 14, 93.3%), with only one late complication reported in the form of pancreatic pseudocysts (n = 1, 6.6%), and no deaths were recorded in this study.

## Discussion

AP was defined by the presence of two of the following criteria: abdominal pain consistent with acute pancreatitis, serum amylase, and/or lipase levels exceeding three times the upper limit of normal and imaging results confirming the diagnosis [1].

Due to the limited duration of our study, we were unable to determine whether the incidence of pediatric acute pancreatitis has increased. However, several recent studies indicate an increase in this incidence over the past few decades, estimated at between one and three cases per 10,000 children. This rise may result from improved awareness, increased diagnoses, referrals to specialized centers, and the emergence of cases in children with other systemic conditions [2,3]. In the literature, the mean age of patients with pediatric acute pancreatitis varies from 7.5 to 14 years.

In our study, the average age was 10 years, which is consistent with other series [4,5]. Although male predominance was evident in our study, acute pancreatitis appears to be a sex-independent condition, as suggested by some studies reporting both male and female predominance [2,4]. Abdominal pain, often epigastric, is the primary symptom of pancreatitis, frequently accompanied by back radiation and a protective posture [6]. Vomiting, either bilious or food-related, is also common, as is general malaise, which varies in prevalence across studies from 9% to 61% [7,8]. Associated signs such as fever, jaundice, tachycardia, and hypotension may accompany episodes of pancreatitis, but are less frequent. Physical examination often reveals abdominal tenderness, primarily in the epigastric region, and abdominal guarding in a significant number of cases [6,9]. In our study, the average consultation delay was 3.5 ± 2.5 days (range 1-11 days).

Consultation delay refers to the interval between symptom onset and hospital presentation. This delay is in line with other studies on pediatric acute pancreatitis. Early presentation is essential to initiate prompt diagnosis and management, which may reduce the risk of complications. Delays in consultation could contribute to disease progression and worse outcomes. Thus, awareness among caregivers and primary healthcare providers of early signs of acute pancreatitis is critical to shorten this delay and improve prognosis [5,8].

In our study, we observed hyperlipasemia in 100% of cases, leukocytosis with neutrophil predominance in 78.5% of patients, and elevated CRP in 100% of cases. These results are higher than those reported in other studies: hyperlipasemia was found in 98-50% of cases, leukocytosis in 53-33.3%, and elevated CRP in 58.8-25% [6,9,10].

Radiologically, abdominal ultrasound revealed abnormalities in 50% of patients, primarily pancreatic hypertrophy (21.4%) and ascites (21.4%), while abdominal CT showed more pronounced anomalies: ascites in 71.4% of cases, enlarged pancreas in 57.1%, and pancreatic necrosis in 50%. Peripancreatic fat infiltration was present in 42.9% of cases, and a pancreatic fracture was observed in 7.1%. Other abnormalities included venous thrombosis (7.1%) and biliary dilation in 14.3%. These results are compared to other studies, where certain anomalies were more or less frequent, particularly necrosis and ascites [6,7].

In this study, the majority of pediatric acute pancreatitis cases were idiopathic in origin (28.6%), a result similar to other research, where this cause varies between 6% and 52.5%. Biliary and traumatic pancreatitis followed, each representing 21.4% of cases, rates that align with previous studies. Drug-induced pancreatitis, primarily due to L-asparaginase, accounted for 7.1%, which is also frequently reported in the literature. Systemic and familial pancreatitis were less common (7.1%), and no metabolic pancreatitis was observed, which is consistent with other studies where these causes remain rare [3,6,7,8].

This study evaluated the performance of various biological markers and severity scores for acute pancreatitis in children. The results show that while some markers, such as lipase and uremia, have good sensitivity, their specificity and predictive value are limited. The severity scores (PAPS, pediatric JPN, Glasgow, Ranson) demonstrated low sensitivity, despite good specificity in some cases. These findings suggest that these diagnostic tools require improvements to better predict the severity of pediatric acute pancreatitis [11,12].

In this study, 57.1% of patients experienced local complications, such as pancreatic necrosis and pseudocysts. Only one case of renal dysfunction was observed (7.1%), with no cardiovascular or respiratory dysfunction or systemic inflammatory response syndrome (SIRS). Compared to other studies, local complications vary from 12.7% to 61.4%, and organ dysfunction is reported between 2% and 11.7%. SIRS is observed in 9.2% to 28.8% of cases in the literature [13].

In our study, medical treatment was based on digestive rest, rehydration, and analgesics. Almost half of the patients received antibiotics, a rate similar to other studies but lower than that reported by others [6,8,10]. PPI treatment was administered to half of our patients, well below the rates reported in the literature [6,8,10]. No surgical treatment was performed, contrary to one study that reported surgical interventions in 6% of cases [6].

In this study, 92.9% of patients had a favorable prognosis, with only one late complication (pancreatic pseudocysts, 7.1%). Pseudocysts are the most common complication of pediatric acute pancreatitis, with an incidence ranging from 2% to 20% [6,10]. No deaths were reported in our series, in contrast to other studies where mortality varies from 2% to 27%, often due to infections in patients with comorbidities such as autoimmune diseases or neoplasms [6,10].

This study has several limitations. First, its retrospective nature may have led to missing or incomplete data. Second, the study was conducted at a single center, which may limit the generalizability of the results.

## Conclusions

AP is a rare but increasingly recognized condition in children, with a broad spectrum of clinical presentations and multiple underlying etiologies. Abdominal pain remains the hallmark symptom, often prompting initial evaluation. Measurement of serum lipase is essential for diagnosis, while imaging, particularly ultrasound and computed tomography (CT), plays a key role in confirming the diagnosis, assessing severity, detecting complications, and guiding follow-up.

Idiopathic and traumatic causes are the most frequent in pediatric cases, followed by biliary, infectious, and drug-related etiologies. The clinical course and prognosis depend largely on the occurrence of local or systemic complications, as well as organ dysfunction.

Management is primarily supportive, including pain control, digestive rest, and rehydration. Surgical or interventional procedures are reserved for selected cases with complications or specific underlying causes. Early diagnosis and appropriate supportive care are crucial to ensure favorable outcomes and prevent long-term sequelae.
